# Epoxy-a-lapachone in nanosystem: a prototype drug for leishmaniasis assessed in the binomial BALB/c - *Leishmania (Leishmania) amazonensis*

**DOI:** 10.1590/0074-02760240115

**Published:** 2024-10-28

**Authors:** Juliana Figueiredo Peixoto, Luiz Filipe Gonçalves-Oliveira, Geovane Dias-Lopes, Franklin Souza-Silva, Carlos Roberto Alves

**Affiliations:** 1 Universidade Católica de Petrópolis, Petrópolis, RJ, Brasil; 2 Fundação Oswaldo Cruz-Fiocruz, Instituto Oswaldo Cruz, Laboratório de Biologia Molecular e Doenças Endêmicas, Rio de Janeiro, RJ, Brasil; 3 Universidade do Estado do Rio de Janeiro, Instituto de Biologia Roberto Alcântara Gomes, Departamento de Ciências Biomédicas e Saúde, Cabo Frio, RJ, Brasil; 4 Fundação Oswaldo Cruz-Fiocruz, Centro de Desenvolvimento Tecnológico em Saúde, Rio de Janeiro, RJ, Brasil; 5 Universidade Iguaçu, Nova Iguaçu, RJ, Brasil

**Keywords:** cutaneous leishmaniasis treatment, combination therapy, epoxy-a-lapachone, microemulsion, meglumine antimoniate

## Abstract

This perspective presents and supports arguments for a new formulation of epoxy-α-lapachone loaded microemulsion (ELAP-ME), a nanosystem, as a prototype drug for the treatment of leishmaniasis. The benefits of ELAP as a multitarget compound, with properties that affect key physiological pathways of *Leishmania* spp. are discussed. ELAP-ME demonstrated efficacy in murine infection models, particularly with the binomial BALB/c-*Leishmania (Leishmania) amazonensis*. Furthermore, it is proposed that the technological maturity of ELAP-ME be classified as Technology Readiness Level 4 (TLR 4) within the context of innovative drugs for American Cutaneous Leishmaniasis (ACL).

At the 16th World Health Assembly in 2007, resolution WHA60.13 was issued, which addressing prospects for controlling leishmaniasis.^1^ This document recognised that infection by parasites of the genus *Leishmania* causes one of the most neglected tropical diseases (NTDs) globally. Leishmaniases are endemic in approximately 99 countries, with over 12 million people infected. Cutaneous leishmaniasis (CL) occurs in 89 countries, while visceral leishmaniasis (VL) affects 80 countries, and both clinical forms are endemic in 71 countries.^2^ These diseases are strongly influenced by factors such as poor housing conditions, lack of financial resources, population displacement, malnutrition, and weakened immune systems of affected individuals, leading to their classification as NTDs.^3^

Despite the significant impact of leishmaniases, treatment options for its various clinical forms remain limited and outdated.^4^ ^-^ ^10^ The search for solutions to control diseases caused by *Leishmania* spp. involves considerable efforts by authorities, research institutes, and health professionals. However, concrete proposals for new drugs with effective control and prevention capabilities are still scarce. This perspective reflects the context highlighted in resolution WHA60.13, which encourages research into leishmaniasis control through the investigation and development of new drugs. This paper presents a critical argument supported by *in vitro* and *in vivo* evidence of the effects of naphthoquinone epoxy-α-lapachone (ELAP) on *Leishmania* spp. in experimental model for CL (Figure).

**Figure:**
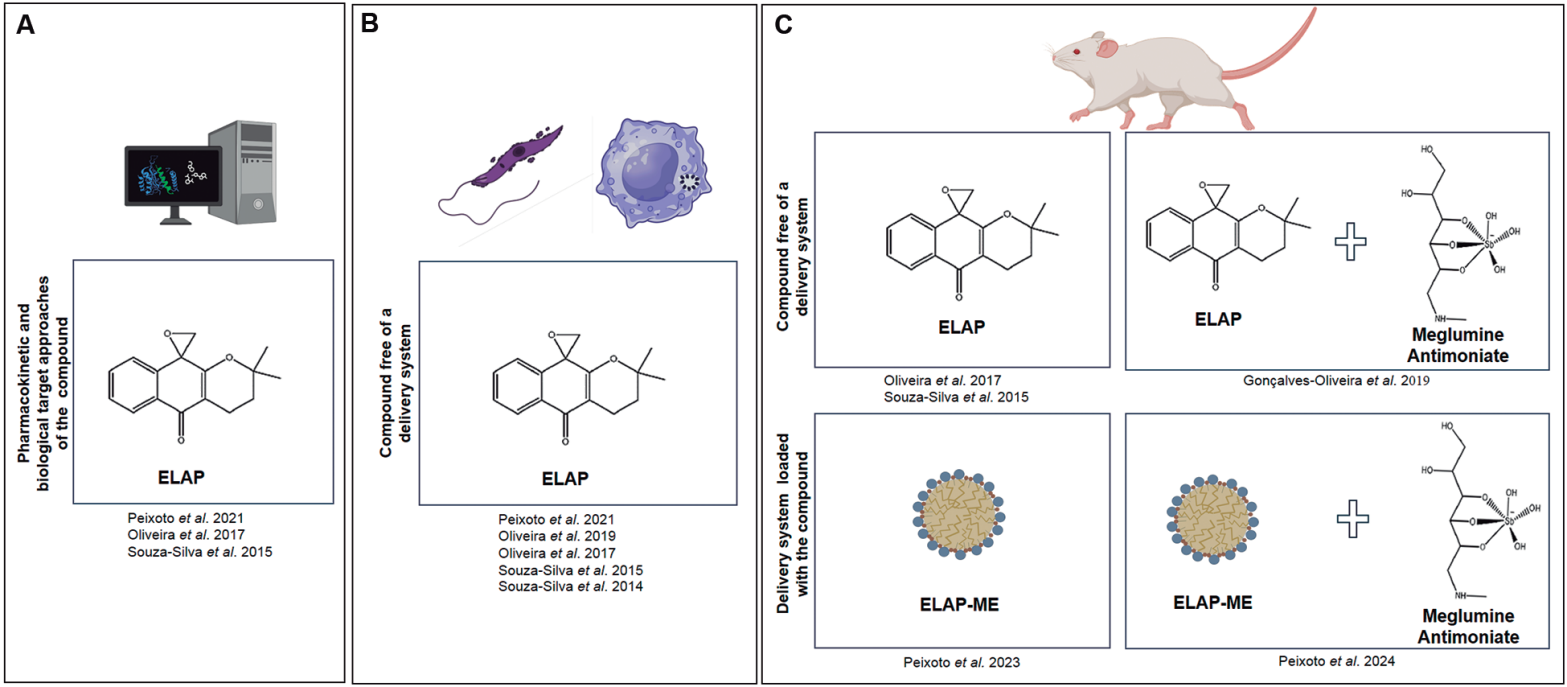
Overview of advances in studies of the epoxy-α-lapachone (ELAP) compound as a prototype drug incorporated into a delivery system. The main steps covered by the ELAP compound as a prototype drug are shown. Throughout studies, potential biological targets and pharmacokinetic parameters have been investigated using *in silico* approaches (A). *In vitro*, investigations of the effects of ELAP on *Leishmania* spp. confirmed its direct action on promastigotes and intracellular amastigotes of *Leishmania (Leishmania) amazonensis* and *Leishmania (Viannia) braziliensis*, as well as murine and human macrophages toxicity (B). The *in vivo* ELAP efficacy also was assessed (C). First, the ELAP free of a delivery system was tested in monotherapy and combination therapy with Meglumine Antimoniate, showing a great leishmanicidal effect in mice infected by *L. (L.) amazonensis*, as well as tissue toxicity. Then, studies of ELAP as a prototype drug progressed by incorporating this compound into an oil-in-water-type microemulsion (ELAP-ME), assessing its efficacy on experimental infection and toxicity by biochemical parameters.

**Evidence of ELAP effect against *Leishmania* spp.: a multitarget compound?**

The development of ELAP follows the expected trajectory for new drug, which generally begins with the selection of candidate molecules through the identification and elucidation of specific targets (Figure A). At this initial stage, *in silico* studies can help elucidate physicochemical parameters such as solubility and selectivity, and contribute to understanding potential mechanisms of action. These assays can be conducted using drug-target interactions, the principle of chemical similarity (similar drugs bind to similar targets), and the principle of molecular biophysics.^11^

Moreover docking simulations proposed the potential of ELAP to form stable complexes with key enzymes in the physiology of *Leishmania* spp. This compound demonstrated the ability to form stable complexes with key enzymes involved in the metabolic pathway, electron transport chain, and lipid metabolism of *L. (Leishmania) amazonensis* and *Leishmania (Viannia) braziliensis*.^12^ *In vitro* assays confirmed the effects of ELAP on *L. (L.) amazonensis* and *L. (V.) braziliensis*, directly affecting promastigotes and amastigotes, as well as exhibiting toxicity towards mammalian cells and tissues^13^ ^,^ ^14^ ^,^ ^15^ (Figure B). Previous evidence indicates that ELAP binds to oligopeptidase B, a serine proteinase from *L. (L.) amazonensis*, by interacting with an S1 binding site.^14^ These molecular interactions reinforce ELAP’s multitarget potential, with ability to inhibit a range of *Leishmania* spp. enzymes, which may influence its effectiveness as a potential drug for leishmaniasis treatment. Furthermore, it is worth noting that other pharmacologically active naphthoquinone derivatives, such as epoxymethyl-lawsone (LAW), which share chemical similarities with ELAP, may exhibit similar mechanisms of action on the parasite’s metabolic pathway, electron transport chain and lipid metabolism. Further studies on LAW are necessary, as previous evidence supports its potential use in leishmaniasis treatment.^15^ ^,^ ^16^ ^,^ ^17^

The development and clinical use of multitarget drugs have proven successful treating complex diseases like leishmaniasis, as demonstrated by the therapeutic efficacy of amphotericin B and miltefosine. These drugs not only act on known mechanisms but also target additional pathways.^18^ Amphotericin B, aside from binding to ergosterol and forming pores in the parasite membrane, may activate the overexpression of several genes, such as the ascorbate peroxidase gene (APx), leading to increased reactive oxygen species and elevated calcium ion levels in the cytoplasm, triggering cell death cascades.^19^ ^-^ ^25^ Miltefosine, besides inhibiting the synthesis of parasite membrane phospholipids, may increase metacaspases production, block cellular replication machinery, elevate calcium ion levels, induce mitochondrial stress, and modulate the host’s immune system.^26^ ^,^ ^27^ ^,^ ^28^ ^,^ ^29^ ^,^ ^30^

Factors such as the biological complexity of *Leishmania* spp., the different clinical forms of leishmaniasis, therapeutic failure, and cases of resistance to current drugs reinforce the need for multitarget approaches in treatment.^31^ Evidence suggests that multitarget compounds with selective actions offer a better balance of efficacy and safety compared to single-target compounds.^32^ This strategy includes polypharmacology, which considers traditional and novel approaches, such as drug combination schemes, where two or more compounds and/or drugs are selected rationally for different targets.^33^ ^,^ ^34^ ^,^ ^35^ ^,^ ^36^ Among the drugs approved by the Food and Drug Administration (FDA) between 2015 and 2017, 21% were multitarget drugs resulting in improved therapeutic effects.^37^ ^,^ ^38^ Given the need for more effective drugs for leishmaniasis treatment, therapies targeting multiple pathways could help prevent resistance and reduce the toxicity. Developing and testing multitarget compounds like ELAP with the potential to interact with key physiological networks of the parasite is crucial.

**A microemulsion loaded with ELAP-ME against *L. (L.) amazonensis* murine infection: a suitable compound delivery system?**

The biological activity of ELAP as a raw material has been effectively assessed, as well as explored its possible mechanism of action.^13^ ^,^ ^14^ ^,^ ^15^ ^,^ ^17^ However, as anticipated for a naphthoquinone derivative, ELAP demonstrated to be very poorly soluble in aqueous solvents typically required for *in vivo* assays. The challenge was to incorporate and maintaining ELAP in a delivery system, to enhance solubility, stability and, consequently, bioavailability to achieve the desired therapeutic effect in animal models. The incorporation of ELAP into a microemulsion type (ME) delivery system, ELAP-ME (100 µg/mL of ELAP), allowed for effective delivery in mouse tissues, maintaining therapeutic concentrations. The development of a ME with adequate droplet size (120 ± 7.7 nm) and stability (over 73 days), was favourable for maintaining the pharmacological effects of ELAP.^39^ This ME, a colloidal dispersion system, enabled stable administration of ELAP, a fat-soluble compound, in a homogeneous mixture of emulsifying components with varying polarities. The characteristics of ELAP-ME offer advantages in terms of absorption and distribution in the body, enhancing its bioavailability.^40^ ^,^ ^41^

Approximately 40% of new compounds under therapeutic investigation have low solubility in water. In addition to MEs, various strategies have been employed to address low solubility, such as solid dispersions, liposomes, solid lipid nanoparticles, and self-emulsifying drug delivery systems.^42^ MEs with similar compositions (Capmul MCM, Labrasol, and PEG 400) have been tested in other treatments. Successful examples include the transdermal anti-inflammatory activity incorporating oleoresin-gum from *Boswellia carterii* ^43^ and self-nano emulsifying systems containing ziprasidone for the treatment of schizophrenia and bipolar disorder.^44^ For curcumin, which suffers from poor absorption, and rapid metabolism, incorporation into nanoparticles has been explored for treating leishmaniasis and other diseases.^45^ ^,^ ^46^ ^,^ ^47^

The development of delivery systems is at the forefront of improving pharmacokinetic properties, efficacy, and reducing toxicity. ELAP-ME absorption studies indicate rapid uptake within 90 minutes after subcutaneous administration, followed by a slow decrease in compound concentration over 12 hours, suggesting distribution to tissues.^39^

**Impact of ELAP-ME on experimental mice infection: effective against the of binomial BALB/c - *L. (L.) amazonensi*s?**

To evaluate the success of leishmaniasis treatment in experimental infection, it is essential to access elements that reveal treatment effectiveness in animal models, along with pharmacokinetic properties and toxicity profiles.^48^ BALB/c mice have been widely used to understand the life cycle, infection process, and host-parasite interactions of *Leishmania* spp., as well as to simulate human infection.^49^ Mice, inbred strains of *Mus musculus*, has been useful for understanding several aspects related to infection by *Leishmania* spp., such as the immunological mechanisms mediating pathogenesis, inflammation profile, therapeutic response, and resistance mechanisms.^50^ These animals have several advantages due to small size, ease of handling, low cost of maintenance, short gestation, numerous offspring, and genomic similarities to humans.^51^

The binomial BALB/c - *L. (L.) amazonensis* has proven beneficial for studying the therapeutic potential of ELAP-ME. Experimental infections in this model are challenging to control, but results indicate a reduction in paw oedema and parasite load at the infection site and in the lymph nodes, highlighting the robustness of the treatment response.^39^ ^,^ ^52^ However, further therapeutic approaches with ELAP-ME are need to achieve optimal infection control, including total reduction of paw oedema, similar to human lesions treated with currently recommended medications.

The combination of ELAP with Glucantime^TM^ (meglumine antimoniate - MA) *in vitro* and *in vivo* assays to control *L. (L.) amazonensis* infection led to better explorations of the efficacy of this treatment regimen (Figure B-C). The choice of MA for the combination trials was due to it being one of the most used drugs in the treatment of leishmaniasis. The use of pentavalent antimonials as first-line drug and the persistence on these compounds for more than 80 years in clinics have provided solid knowledge on the use of these compounds with important recommendations for the clinical monitoring of patients with American Cutaneous Leishmaniasis (ACL). Furthermore, studies have highlighted alternative treatment regimens, such as the use of low doses (5 mg Sb^5+^/kg/day), intermittent regimens, the development of a standard procedure for intralesional administration, and new processes for the production of a generic formulation.^53^ ^-^ ^58^ These strategies have proven to be effective and reduce toxic effects, but they remain costly. Other efforts to improve antimonial chemotherapy have been proposed, focusing on the chemical structure, mechanisms of action, as well as preparation methods and different formulations of antimonials.^59^ ^,^ ^60^ Approaches involving pentavalent antimonials in combination with other drugs, such as imiquimod, allopurinol and immunotherapy have been explored, but some results are still controversial.^61^ ^,^ ^62^ ^,^ ^63^ ^,^ ^64^ ^,^ ^65^

Despite the challenges presented by treatment with pentavalent antimonials, all these efforts aim to maintain antimonial chemotherapy, and it is plausible to suggest that replacing it in the short, medium, or long term will be difficult.^53^ Following the trend of sustaining the use of these compounds in clinical practice, previous studies have demonstrated that the experimental treatment strategy combining ELAP-ME with MA may be a viable alternative, as it is maintains efficacy with reduced doses and a lower toxicity profile.^52^

The combined treatment of ELAP-ME and MA in different proportions led to the best effects in the binomial BALB/c - *L. (L.) amazonensis*.^52^ It is worth noting that it is possible to achieve similar efficacy at lower doses compared to MA monotherapy when used at a dose equivalent to that recommended by the WHO for ACL patients.^66^ ^,^ ^67^ Another relevant aspect is the ability of ELAP-ME to achieve therapeutic efficacy in a shorter course of treatment and with a lower dosage when compared to previous studies using unformulated ELAP.^17^

Combination assays with other compounds incorporated into MEs have been tested *in vitro* and *in vivo* for ACL. The incorporation of amphotericin B and terbinafine in MEs showed greater efficacy in reducing the lesion size in BALB/c mice infected with *L. (L.) major* and the parasite load when compared to raw drugs. Combination regimens proved more effective and less toxic compared to drugs in monotherapy.^68^ ^,^ ^69^ A systematic review covering 14 preclinical studies compared the efficacy of combination regimens with monotherapy in experimental models (mice, hamsters, or dogs) in VL. In these studies, 23 drugs were tested in 40 different combinations, with most of these regimens resulting in reduction in effective dose, cost, and treatment time, in addition to improving parasitological control.^70^

Furthermore, it is necessary to consider that the effectiveness of ELAP-ME may have been influenced by the chosen administration route, another key factor in the development of a drug. Except for miltefosine, a first-line oral drug in Brazil, pentavalent antimony derivatives, and other therapeutic options are administered parenterally, whether intramuscularly, intravenously or intralesional.^67^ Although parenteral administration is an undesirable route for patients and difficult for them to adhere to, the purpose in the mouse model was to maintain the efficacy of MA and ELAP observed in previous studies, prioritising the subcutaneous route.

Other advantages of the ELAP-ME combination regimen with MA were observed, such as lower dosages of renal (creatine kinase: CK and nitrogenous urea: Urea/BUN) and hepatic (alanine aminotransferase: ALT) and aspartate aminotransferase: AST) serum parameters, suggesting lower toxicity in these organs when compared to MA monotherapy at two doses per week.^52^ Indeed, one of the essential requirements for drug development is that minimal or mild adverse effects are desirable.^53^ This is a significant factor that discourage some studies on new molecules that, despite being effective, demonstrate toxicity *in vitro* and *in vivo models* and, thus do not advance to the prototype drug stage for clinical testing.

It is important to note that while some reports in the literature describe kidney changes due to *Leishmania* spp. infection in humans even before treatment begins.^71^ ^,^ ^72^ the renal toxic effects caused by Glucantime™ treatment have been well-documented for decades. These effects include acute renal failure, acute kidney injury, acute tubular necrosis, interstitial nephritis, and reduction of the glomerular filtration rate have been reported.^72^ ^,^ ^73^ ^,^ ^74^ Changes in the liver function have also been reported with this medication, such as elevated serum enzymes levels, liver failure, and hepatitis.^74^ ^,^ ^75^ Therefore, the combination regimens of ELAP-ME with MA proposed in previous studies may represent a promising approach to ACL treatment.

Technological maturity of epoxy-a-lapachone as a promising drug for ACL: a prototype drug?

Regarding the problem of treatments currently available for leishmaniasis, the search for new effective drugs which are less toxic and do not allow inducing parasite resistance has been essential. However, the planning, synthesis, and development of new drugs are typically high-cost processes (approximately US$ 2.6 billion) and take a long time (10 to 15 years, on average).^76^ ^,^ ^77^ ^,^ ^78^ Several steps are required for an active compound to be approved for clinical use, from preclinical evaluations (*in vivo* studies in animal models) to clinical trials (on humans) carried out in four successive phases: Phase I (initial phase, assessment of drug safety in healthy volunteers); Phase II (evaluation of the efficacy and safety of the drug in a larger group of patients with the target disease and dose determination); Phase III (confirmation of efficacy and safety in a slightly large group of patients with the disease, comparison of the drug with standard treatment or placebo) and Phase IV (continuous monitoring of efficacy and long-term safety with the drug already approved for use), which must follow all protocols defined by the health authorities (Brazilian Health Regulatory Agency - ANVISA - Collegiate Board Resolution nº 09, of February 20, 2015, which outlines regulations for conducting clinical trials with medicines in Brazil). The ELAP compound has been following this trajectory and remains in the context of preclinical studies, with noticeable evolution in recent decades regarding its pharmacological potential in controlling infections caused by *Leishmania* spp., as well as in studies with *Trypanosoma cruzi* and *Plasmodium* sp.^79^

The stages of discovery and development of ELAP as a new drug show how different areas of knowledge contribute to technological development, from biology, chemistry, and pharmaceutical sciences to mathematical modelling and computer sciences.^48^ The body of evidence from preclinical ELAP assays conducted over the last nine years on *Leishmania* spp. provides the information necessary to guide the prototype drug toward a new stage of technological maturity.^80^ TRL1-TRL3 levels were achieved in studies carried out with raw ELAP, demonstrating the feasibility of using this compound as a possible drug for this parasite.^14^ ^,^ ^16^ ^,^ ^17^ The set of results obtained so far allows the inference of a TRL4 level of technological maturity for ELAP, as they include experimental analyses of efficacy (TRL4A),^39^ ^,^ ^52^ and initial *in vivo* toxicity studies (TRL4B)^52^ following the incorporation of this compound into a nanosystem. However, these steps must certainly comply with good laboratory practices for the development of medicine (Good Laboratory Practices - GLP).

As desired for ELAP-ME, as new drugs progress through the development stages, the likelihood of achieving regulatory approval increases.^81^ To date, only the compounds LXE408 and GSK3494245, two selective proteasome inhibitors, are in clinical phase studies for the treatment of VL, administered orally.^82^ ^,^ ^83^ Therefore, it is important to consider that, despite the level of technological maturity achieved through the development of a delivery system for the ELAP, essential steps must still be taken to complete the preclinical and clinical stages to become a medicine and obtain approval. Among these steps, it is recommended to conduct more robust toxicity and safety studies to confirm the results achieved so far, accordingly ANVISA guidelines through the Guide for Conducting Non-Clinical Toxicology and Pharmacological Safety Studies Necessary for the Development of Medicines.^84^

The research efforts presented in this perspective exemplify the convergence of expertise required to conduct studies aimed at developing new drugs. They represent what is expected of essential technical-scientific autonomy in the innovation of drugs for leishmaniasis, following public health policy recommendations on drug research and development for neglected diseases. Finally, the results presented position ELAP-ME as a prototype of a new drug to treat leishmaniasis, aligning clearly with the guidelines recommended in the Innovation Law (Law 10973/2004, regulated by Decree 5563/2005) in the field of biotechnology and pharmaceutical sciences.

In conclusion

The development of new drugs with desirable efficacy and acceptable toxicity for leishmaniasis is a challenge for global public health since there are limited and outdated treatment options, which cause several adverse effects. In turn, the launch of a new medicine for leishmaniasis must adhere to standards of quality, efficacy, and safety for pharmaceutical products. In this context, ELAP-ME is proposed as a prototype drug at the level of technological maturity TRL-4, as it has advanced through analytical and laboratory studies. Furthermore, there is evidence to suggest that ELAP is a multitarget compound, which can interact with important targets in the physiological and biochemical pathways of *Leishmania* spp., such as proteolysis, energy metabolism, electron transport chain, and lipid biosynthesis. This is a desirable feature for treating multifactorial diseases such as leishmaniasis, which result from factors such as parasite polymorphism, and the host’s immune and genetic background, making it essential for drug actions to target multiple sites within the parasite.

The recommendation of ELAP-ME as an acceptable delivery system is supported by the pharmacotechnical strategy that allowed the development of an oil-in-water ME type delivery system, with sufficient stability for use *in vivo* tests of efficacy and toxicity. Moreover, this nanosystem proved effective in controlling experimental infection in the binomial BALB/c - *L. (L.) amazonensis*, a well-established model, demonstrating the efficacy and toxicity of ELAP-ME in monotherapy or in combination with MA. Combined treatment with MA provides the best therapeutic efficacy when compared to ELAP-ME monotherapy showing reduced damage to kidney and liver functions in mice.

Further studies on the efficacy and safety of using combined ELAP-ME regimens with MA or other drugs should be considered, including tests in different animal models for ACL. Additionally, new routes of administration of ELAP-ME may be evaluated to address the limitations and drawbacks of parenteral treatment. Good manufacturing practices for ELAP-ME must be followed to ensure that this pharmaceutical product is of high quality.
